# Gene expression of lactobacilli in murine forestomach biofilms

**DOI:** 10.1111/1751-7915.12126

**Published:** 2014-04-04

**Authors:** Clarissa Schwab, Alexander Tøsdal Tveit, Christa Schleper, Tim Urich

**Affiliations:** 1 Division of Archaea Biology and Ecogenomics, Department of Ecogenomics and Systems Biology, University of ViennaWien, Austria; 2 Department of Arctic and Marine Biology, University of TromsøTromsø, Norway

## Abstract

Lactobacilli populate the gastro-intestinal tract of vertebrates, and are used in food fermentations and as probiotics. Lactobacilli are also major constituents of stable biofilms in the forestomach of rodents. In order to investigate the lifestyle of these biofilm lactobacilli in C57BL/6 mice, we applied metatranscriptomics to analyse gene expression (assessed by mRNA) and community composition (assessed by rRNA). *Lactobacillales* were the major biofilm inhabitants (62–82% of rRNA reads), followed by *Clostridiales* (8–31% of rRNA reads). To identify mRNA transcripts specific for the forestomach, we compared forestomach and hindgut metatranscriptomes. Gene expression of the biofilm microbiota was characterized by high abundance of transcripts related to glucose and maltose utilization, peptide degradation, and amino acid transport, indicating their major catabolic and anabolic pathways. The microbiota transcribed genes encoding pathways enhancing oxidative stress (glutathione synthesis) and acid tolerance. Various pathways, including metabolite formation (urea degradation, arginine pathway, γ-aminobutyrate) and cell wall modification (DltA, cyclopropane-fatty-acyl-phospholipid synthase), contributed to acid tolerance, as judged from the transcript profile. In addition, the biofilm microbiota expressed numerous genes encoding extracellular proteins involved in adhesion and/or biofilm formation (e.g. MucBP, glycosyl hydrolase families 68 and 70). This study shed light on the lifestyle and specific adaptations of lactobacilli in the murine forestomach that might also be relevant for lactobacilli biofilms in other vertebrates, including humans.

## Introduction

The genus *Lactobacillus* encompasses a diverse group of bacteria from the *Firmicutes* phylum that act as starter cultures in fermented foods, are detected in the gastrointestinal tract (GIT) of humans and animals, and are applied for their health-promoting effects as probiotics (Claesson *et al*., 2007). It is much less known that strains of *Lactobacillus* are autochthonous to the proximal parts of the GIT of rodents, birds, pigs and horses forming stable biofilms (Walter, 2008). Lactobacilli adhere to the non-glandular squamous stratified epithelium lining the forestomach of rodents, the crop of birds, the oesophagus of pigs and the non-glandular stomach of horses (Savage, 1972; Fuller *et al*., 1978; Yuki *et al*., 2000; Walter *et al*., 2011).

One of the best characterized species of these biofilms is *Lactobacillus reuteri*, which is also able to persist in the human gut and is frequently recovered from cereal fermentations (Gänzle and Schwab, 2009b; Walter *et al*., 2011). Population genetics revealed that strains of *L. reuteri* cluster into host-specific clades (Oh *et al*., 2010; Su *et al*., 2012). Whole genome comparison by Frese and colleagues (2011) and Su and colleagues (2012) indicated that the genomic potential of rodent and human *L. reuteri* differs, and that cereal isolates have traits of both rodent and human strains. For example, only rodent isolates possess a urease cluster, while human isolates are capable of producing reuterin and 1, 2-propanediol via the activity of the *pdu-cbi-cob-hem* cluster (Frese *et al*., 2011; Su *et al*., 2012).

A similar scheme of host–microbe co-evolution was observed for *L. johnsonii* (Buhnik-Rosenblau *et al*., 2012). Mouse forestomach colonization of *L. johnsonii* isolates is dependent on strain origin: a rodent isolate (100-33) persisted in RLF-mice similar to *L. reuteri* (log 9 cfu g^−1^ in the forestomach), while numbers of human gut (NCC533) or blood (ATCC33220) isolates maximally reached log 7 cfu g^−1^ and decreased within days in conventional and antibiotic-treated mice (Denou *et al*., 2008; Tannock *et al*., 2012).

In comparison to the autochthonous strains of *L. reuteri* and *L. johnsonii*, *L. plantarum* is considered allochthonous as it does not persist in mice with complete gut microbiota after single injection and quickly transits through the GIT (Marco *et al*., 2007). Nevertheless, several studies on *L. plantarum* determined gene expression and identified key genes transcribed during transition through the lower GIT using microarrays or *in vivo* expression technology (IVET). Some genes, e.g. sugar phosphotransferase system (PTS), copper transporting ATPase, were also transcribed *in vivo* by *L. johnsonii* NCC533 (Bron *et al*., 2004; Denou *et al*., 2007; Marco *et al*., 2007; 2009).

Lactobacilli-free reconstituted mice (RLF-mice, Tannock *et al*., 1988), complex microbiota mice pre-treated with antibiotics and force-fed with the strains of interest, and germ-free re-associated mice are frequently applied tools to investigate *Lactobacillus* lifestyle in the murine intestine (Bron *et al*., 2004; Walter *et al*., 2005; 2007; 2008; Denou *et al*., 2007; 2008; Marco *et al*., 2007; 2009; Frese *et al*., 2011; Tannock *et al*., 2012). In contrast, little is known about the natural autochthonous biofilm microbiota residing in the murine forestomach. Studies on natural communities can give insights into community structure and function of the forestomach biofilms and can validate the results obtained from model systems commonly applied. We, therefore, chose a metatranscriptomic approach to determine community composition and activity in the forestomach epithelium of widely used C57BL/6 mice. With this study, we aimed to find answers to the following questions: Who resides in natural biofilms on stratified squamous epithelium? What are the interactions and metabolic pathways of the community? Does host niche affect gene expression?

## Results and discussion

We applied metatranscriptomics, which was previously shown to predict activity level of bacterial communities (Helbling *et al*., 2012), to concurrently investigate community composition and function of autochthonous forestomach biofilms in mice. Metatranscriptomic data derived from pooled hindgut contents of caecum and colon were used to enable detection of host niche-specific impacts on gene expression. Metatranscriptomics of five individual forestomach biofilm microbiotas resulted in datasets consisting of 0.5–4.8 Mio sequences, with the majority (92.3–98.4%) being ribosomal RNA (Table S1). The metatranscriptomic datasets of the hindgut microbiotas contained between 3.2 and 13.3 Mio sequences. The utilization of two different sequencing techniques should otherwise not result in considerable data bias, as cDNA synthesis was identical, and the DNA template fragment length and the length of the generated sequences were similar due to the usage of overlapped paired-end reads for Illumina (∼ 160 versus ∼ 210 bp; Table S1). Due to the different sizes of the datasets from IonTorrent and Illumina sequencing (Table S1), we focused on transcripts that were significantly higher transcribed in the IonTorrent derived metatranscriptomes from the forestomachs. It has to be acknowledged that sequencing depth is an issue with metatranscriptomic analysis. However, in comparison with other transcriptomic approaches (e.g. microarray and IVET), the resolution power of metatranscriptomics is high. This is, to our best knowledge, the biggest metatranscriptomic study about the lifestyle of lactobacilli in the murine intestine to date.

### The forestomach biofilm microbiota

**Fig. 1:**
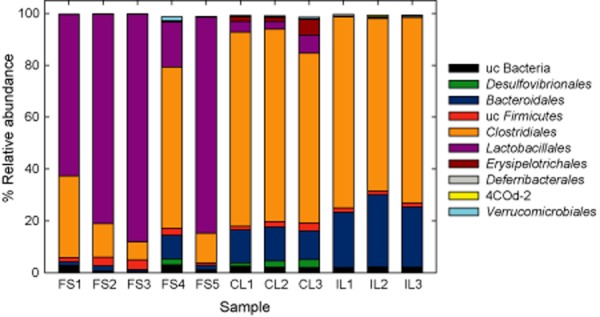
Forestomach and hindgut bacterial communities. Bacterial orders present in the murine forestomach and in the hindgut based on relative abundance of 16S rRNA transcripts. Shown are the bacterial communities of five forestomachs (FS1-FS5) and of six hindgut communities (CL1-3, IL1-3). uc, unclassified; 4COd-2 *Cyanobacteria*-like lineage.

Community composition was determined using 16S rRNA transcripts from the metatranscriptomes. We consider this a measure of relative abundance of bacterial groups even though the rRNA content does not necessarily reflect cellular abundance and is affected in complex ways by the physiological state of the cell. *Lactobacillales* were the dominant bacterial order (62–82%) in the forestomach (FS), with the exception of FS4 where *Clostridiales* prevailed (Fig. 1). Quantitative polymerase chain reaction (PCR) targeting the 16S rRNA gene confirmed the high proportion of lactobacilli in the forestomach bacterial community (Table S2). In FS1-3 and FS5, *Clostridiales* represented between 8% and 31% of the community and consisted mainly of the families *Lachnospiraceae* (approximately 73%) and *Ruminococcaceae* (approximately 17%). Relative abundance of *Bacteroidales* fluctuated between 1% and 2%, with the exception of FS4 (9%). *Desulfovibrionales* (0.12–2.69%), 4COd-2 (up to 0.04%) and *Deferribacterales* (mainly the genus *Mucispirillum*, up to 0.21%) were repeatedly retrieved from forestomach and were also present in the hindgut. In agreement to the complex community determined in this study, Fuller and colleagues (1978) reported a phenotypically diverse, lactobacilli-dominated biofilm in the oesophagus of pigs. In contrast to the forestomach, *Clostridiales* and *Bacteroidales* were the dominant bacterial orders in the hindgut. The proportion of *Lactobacillales* was much lower, fluctuating between 0.5% and 4% (Fig. 1).

**Fig. 2:**
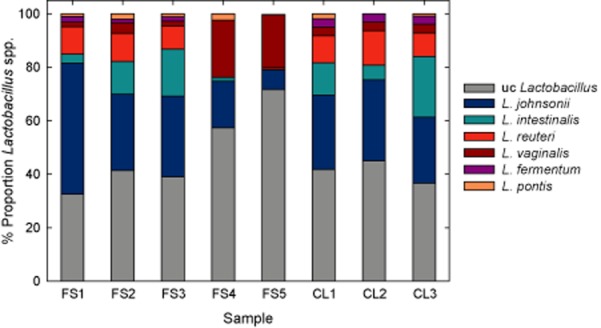
Proportion of *Lactobacillus* species present in the forestomach and hindguts. *Lactobacillaceae* 16S rRNA transcripts were assigned to a modified *Lactobacillus*-type strain database using BlastN (see *Experimental procedures* for details). On average, 58% of all *Lactobacillaceae* 16S rRNA transcripts could be assigned on species level. uc, unclassified.

To investigate the *Lactobacillus* species composition in more detail, we compared the identified *Lactobacillaceae* 16S rRNAs against a references database of *Lactobacillus*-type strains using BlastN. In a prior simulation test, we validated the reference database and analytical approach (see *Experimental procedures* for details). On average, 58% of the *Lactobacillaceae* rRNAs could be assigned to a species, with the exception of FS5 (28%). All detected *Lactobacillus* species belonged to the subgroups of *L. johnsonii/acidophilus* or *L. reuteri* (Fig. 2, Canchaya *et al*., 2006; Felis and Dellaglio, 2007). In three forestomachs (FS1-3), *L. johnsonii* and *L. intestinalis* constituted more than 70% of the classified *Lactobacillus* population, while in FS4 and FS5, *L. vaginalis* was predominant (Fig. 2). *L. johnsonii* and *L. vaginalis* were detected in all forestomachs, whereas *L. reuteri*, *L. fermentum*, *L. pontis* and *L. intestinalis* were irregularly recovered. Only *Lactobacillus* species detected in the forestomach were also present in the hindgut (Fig. 2).

While the co-colonization of the forestomach by *L. reuteri* and *L. johnsonii* has been reported before (Tannock *et al*., 2012), the presence of *L. vaginalis*, sometimes even outnumbering all other *Lactobacillus* species, was unexpected. *Lactobacillus vaginalis* was originally isolated from the human vagina, which is also lined by squamous stratified epithelium (Embley *et al*., 1989). Surprisingly, *L. pontis*, which was originally isolated from sourdough (Vogel *et al*., 1994) and belongs to the *L. reuteri* subgroup (Canchaya *et al*., 2006; Felis and Dellaglio, 2007), was frequently detected. It cannot be excluded that *L. pontis* is allochthonous and stemmed from feed as it has not previously been detected in mice. However, for *L. reuteri*, it is known that rodent and human strains can colonize both cereal and intestinal habitats (Walter *et al*., 2011; Gänzle and Schwab, 2009a; Su *et al*., 2011).

### Gene expression in the forestomach and hindgut

We obtained between 22.255 and 144.454 putative mRNAs from forestomach biofilms and up to 755.736 from the hindgut (Table S1). On average, approximately 30% of these could be functionally annotated with the SEED subsystems (Mitra *et al*., 2011, Table S1). In FS1-3 and 5, between 44% and 87% of the mRNA reads were taxonomically assigned to *Lactobacillales*, whereas in FS4 lactobacilli contributed only 13% of mRNA reads (Table S1). This difference was in accordance with the rRNA-derived community composition (Fig. 1).

**Fig. 3:**
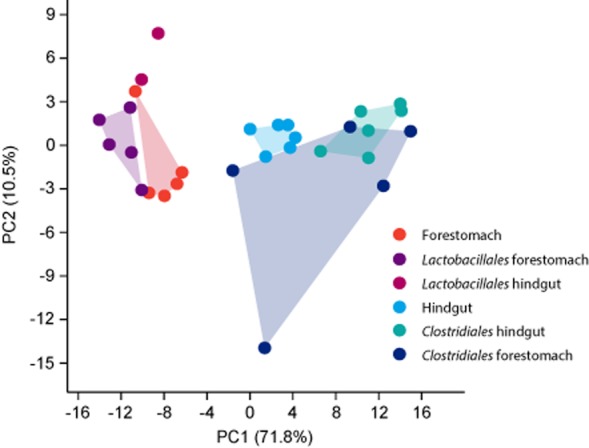
Host niche-dependent gene expression. Principal component analysis based on relative abundance of SEED categories in the forestomach and the hindgut of either the entire community (hindgut, forestomach) or of *Lactobacillales* and *Clostridiales* residing in forestomach or hindgut. Only the two hindgut metatranscriptomes that yielded more than 1000 transcripts assigned to the *Lactobacillales* were included in the analysis.

SEED functional category profiles differed depending on location (forestomach versus hindgut, Fig. 3) and were generally defined by the transcription pattern of the prevailing bacterial orders (*Clostridiales* in hindgut and *Lactobacillales* in forestomach) (Fig. S1). Location within the GIT (forestomach versus hindgut) did not impact the gene expression pattern of *Lactobacillales* and *Clostridiales* to a large extent at least when analysed on the most general level of the SEED functional categories (Fig. 3). However, this might be different within more specific metabolic categories. As only two hindgut samples yielded enough *Lactobacillales* mRNA reads for functional analysis, we strengthened these findings including two additional samples obtained from Tyk2^-/-^ mice on a C57BL/6 background with *n* = 1504 and *n* = 1071 *Lactobacillales* mRNA reads (Fig. S2).

In contrast to the results obtained here, transcription pattern of the human gut isolate *L. johnsonii* NCC355 varied in different segments of the GIT and was very low in the colon as determined using microarrays (Denou *et al*., 2007). This strain is not autochthonous to the murine intestine, which might explain the differences in gene expression. Nevertheless, *L. johnsonii* NCC533 (Denou *et al*., 2007) transcribed the highest numbers of genes in the murine forestomach, pointing at the forestomach as its preferred location during its temporary existence in the mouse GIT.

### Substrate utilization and metabolic network in the forestomach

*Lactobacillus johnsonii* and *L. reuteri* lack biosynthetic pathways for amino acids, purine nucleotides and co-factors (Pridmore *et al*., 2004; Frese *et al*., 2011; Guinane *et al*., 2011). The higher transcript abundance of a number of proteins related to amino acid transport and degradation compared with the hindgut indicated their importance as growth requirements for the biofilm (Table 1). Additionally, Pfam domains indicative of glutamate dehydrogenases were significantly enriched in the forestomach. Glutamate dehydrogenase transforms glutamate to α-ketoglutarate, which increases amino acid conversion as it acts as amino acceptor in transamination reactions (Gänzle *et al*., 2007).

**Table 1:** Pfam categories that are significantly and at least tenfold more abundant in the forestomach than in the hindgut

Functional category	Pfam	Predicted function	% Relative abundance	Fold higher than hindgut	*P*-value
Metabolism					
ABC transporter	EscB (PF05975)	Bacterial ABC transporter	0.020 ± 0.013	nd	0.003
	OprD (PF03573)	Outer membrane porin, OprD family	0.058 ± 0.067	nd	0.030^a^
	OpuAC (PF04069)	Compatible solute binding protein of ABD transporter	0.033 ± 0.016	14	0.001
Sugar transport	Sugar_transport (PF00083)	Putative glucose uptake	0.038 ± 0.030	84	0.013
	Sugar-bind (PF04198)	Putative sugar binding domain	0.114 ± 0.136	34	0.037^a^
Maltose utilization	Glyco_hydro_65C^1^ (PF03633)	Maltose phosphorylase	0.036 ± 0.043	64	0.04^a^
	Glyco_hydro_65 M^2^ (PF03632)		0.168 ± 0.111	43	< 0.001
	Glyco_hydro_65N^3^ (PF03636)		0.068 ± 0.064	41	0.031
Other carbohydrate utilization	Glucosaminidase (PF01832)	Mannosyl-glycoprotein endo-beta-N-acetylglucosaminidase	0.064 ± 0.036	37	0.002
	Glyco_hydro_47 (PF01532)	Alpha-mannosidase (inverting)	0.012 ± 0.012	nd	0.030
Pentose phosphate pathway	G6PD_C^1^ (PF02781)	Glucose-6-phosphate dehydrogenase	0.044 ± 0.025	23	0.003
	G6PD_N^3^ (PF00479)		0.042 ± 0.039	79	0.03
	XFP (PF3894)	D-xylulose 5-phosphate/D-fructose 6-phosphate phosphoketolase	0.481 ± 0.423	30	0.023
	XFP_C^1^ (PF9363)		0.372 ± 0.262	27	0.008
	XFP_N^2^ (PF9364)		1.006 ± 1.163	50	0.032^a^
Amino acid and peptide uptake and metabolism	AA_permease (PF00324)	Amino acid uptake	0.092 ± 0.083	16	0.030
	AA_permease_2 (PF13520)	Amino acid uptake	0.380 ± 0.356	43	0.029
	Peptidase_C1_2 (PF03051)	Peptidase C1-like	0.669 ± 0.386	20	0.003
	Peptidase_C69 (PF03577)	Peptidase	0.656 ± 0.374	19	0.003
	Peptidase_M1 (PF01433)	Peptidase	0.089 ± 0.064	116	0.007
	Peptidase_S15 (PF02129)	X-Pro dipeptidyl-peptidase	0.018 ± 0.014	13	0.019
	A1_Propeptide (PF07966)	Endopeptidase propeptide	0.048 ± 0.043	nd	0.022
	Beta-lactamase2 (PF13354)	Beta-lactamase enzyme family	0.019 ± 0.016	16	0.027
	Asp (PF00026)	Aspartyl protease	0.103 ± 0.115	nd	0.027^a^
Amino acid conversion	Bac_GDH (PF05088)	Glutamate dehydrogenase	0.016 ± 0.015	nd	0.028
Stress tolerance					
Urea uptake and degradation	AmisS_Urel (PF02293)	Urea channel/amide transporter	0.653 ± 0.464	242	0.007
	Urease_alpha (PF00449)	Urease (α-, β-, γ-subunit)	0.460 ± 0.413	22	0.027
	Urease_beta (PF00649)		0.316 ± 0.312	32	0.039
	Urease_gamma (PF00547)		0.525 ± 0.436	41	0.017
	UreD (PF01774)	Urease accessory protein	0.030 ± 0.023	22	0.015
	UreE_C^1^ (PF05194)	Urease accessory protein	0.018 ± 0.013	13	0.011
	UreE_N^3^ (PF02814)	Urease accessory protein	0.018 ± 0.013	nd	0.043^a^
	UreF (PF01730)	Putative activator of urease	0.045 ± 0.025	84	0.002
	Aminohydro_1 (PF01979)	Metal dependent hydrolase superfamily	0.446 0.425	12	0.041
Acid stress	Glutaminase (PF04960)	Glutamine + H_2_O→ Glutamate + NH_3_	0.086 ± 0.044	3	0.011
	Pyridoxal_deC (PF00282)	Glutamate decarboxylase	0.180 ± 0.137	10	0.017
Acid stress/biofilm formation	DltD_C^1^ (PF04914)	Biosynthesis of D-alanyl-lipoteichoic acid	0.040 ± 0.05	368	0.041^a^
	DltD_M^2^ (PF04918)		0.009 ± 0.017	22	ns
	DltD_N^3^ (PF04915)		0.019 ± 0.025	nd	0.044^a^
Acid stress	CMAS (PF02353)	Cyclopropane-fatty-acyl-phospholipid synthase	0.084 ± 0.071	137	0.017
Oxygen tolerance	Glu_cys_ligase (PF04262)	Glutamate-cysteine ligase	0.007 ± 0.006	26	0.020
	GSH-S_ATP (PF02955)	Glutathione synthetase	0.006 ± 0.007	nd	0.039
	GSHPx (PF00255)	Glutathione peroxidase	0.018 ± 0.007	10	< 0.001
	GST_N_3	Glutathione S-transferase	0.018 ± 0.007	85	< 0.001
General stress	Usp	Universal stress protein	0.163 ± 0.062	14	< 0.001
Adhesion/biofilm formation					
Miscellaneous	MucBP (PF06458)	Mucus binding protein	0.308 ± 0.287	169	0.03
	Rib (PF08428)	Mucus binding protein	0.596 ± 0.236	84	< 0.001
	SLAP (PF03217)	Bacterial surface layer protein	0.057 ± 0.073	533	0.041^a^
	YSIRK_signal (PF04650)	Bacterial surface proteins	0.062 ± 0.044	144	0.007
	Glyco_hydro_68 (PF02435)	Levansucrase/invertase	0.011 ± 0.012	50	0.029^a^
	Glyco_hydro_70 (PF02324)	Glucansucrase	0.025 ± 0.030	73	0.037^a^
	DSBA (PF01323)	Introduction of disulfide bonds (periplasmatic)	0.081 ± 0.075	49	0.029
	DSBB (PF02600)	Introduction of disulfide bonds (membrane bound)	0.008 ± 0.008	nd	0.043
	PEPcase (PF00311)	Phosphoenolpyruvate carboxylase	0.033 ± 0.013	98	< 0.001
	Aldedh (PF00171)	Dehydrogenase of aldehyde compounds	0.378 ± 0.199	24	0.001
	E1_dh (PF00676)	Dehydrogenase E1 component	0.087 ± 0.069	56	0.013
	FMN_dh (PF01070)	FMN dependent dehydrogenase	0.063 ± 0.045	18	0.010
	Amidinotransf (PF02274)	amidinotransferase	0.248 ± 0.240	11	0.047
	NAD_binding_10 (PF13460)	NAD binding domain	0.055 ± 0.045	46	0.016
	NAD_binding_2 (PF03446)	NAD binding domain	0.186 ± 0.188	18	0.046
	NADH5_C (PF06455)	C-terminal region of several NADH dehydrogenases	0.026 ± 0.027	123	0.039
	PAS_10 (PF13596)	PAS domain/signal sensor	0.039 0.024	360	0.003

a. One-tailed t-test, all unmarked two-tailed t-test; nd, not detected in the hindgut;

1 C-terminal domain;

2 central catalytic domain;

3 N-terminal domain.

**Fig. 4:**
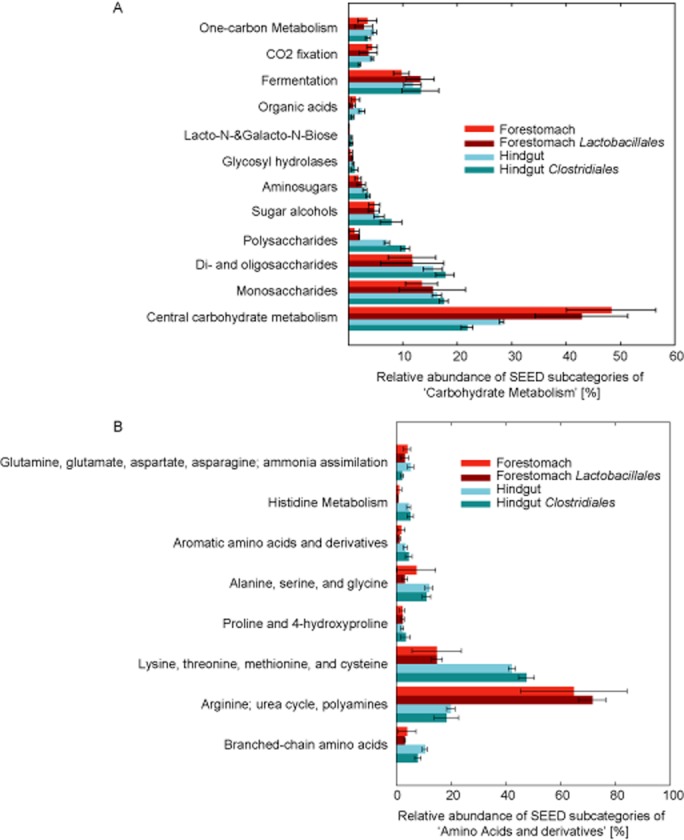
Carbohydrate and amino acid metabolism in forestomach and hindgut. Relative abundances of transcripts assigned to the SEED subcategories of ‘carbohydrate metabolism’ (A) and utilization of ‘amino acids and derivatives’ (B). Shown are either all transcripts recovered from forestomach or hindgut, or transcripts from the forestomach and hindgut assigned to *Lactobacillales* or *Clostridiales* respectively.

With few exceptions, lactobacilli are also not capable of degrading and transporting oligo (*n* > 4) – and polysaccharides (Gänzle and Follador, 2012). Accordingly, the high proportion of transcripts for ‘central carbohydrate metabolism’ (Fig. 4A), which are mainly associated with glucose utilization, together with the high abundance of Pfam domains for a glucose transporter, maltose phosphorylase (key enzymes of the pentose phosphate pathway) and lactate dehydrogenase (Table 1, Table S3), were indicative of glucose and maltose as preferred carbohydrate sources and the major catabolic pathways for their utilization in the forestomach biofilm microbiota.

In contrast, the higher abundance of transcripts for ‘polysaccharides’ and ‘aminosugars’ (Fig. 4A) in hindgut metatranscriptomes, including glycosyl hydrolases involved in cellulose (GH48) and mucin (GH3, GH2C) degradation (Table S3, Schwab *et al*., 2014), indicated mucus and cellulose as possible substrates for the hindgut microbiota. That observation agrees with the transcription of PTS systems for mannose, cellobiose and n-acetylglucosamine by *L. plantarum* and *L. johnsonii* NCC533 during GIT transit (Bron *et al*., 2004; Denou *et al*., 2007).

Relative abundance of *Clostridiales* transcripts assigned to the subcategories of ‘carbohydrate metabolism’ in the forestomach strongly correlated to proportions to those subcategories observed in the hindgut (Pearson correlation, *P* < 0.01), indicating that the *Clostridiales* might also contribute to polysaccharide degradation in the forestomach. *Clostridiales*, which could be introduced to the forestomach via coprophagy, can also utilize lactate, the major fermentation product of lactobacilli, to produce butyrate (Duncan *et al*., 2004). Within the *Clostridiales* population transcriptome, we recovered few transcripts of clostridial lactate dehydrogenase converting lactate to pyruvate (Belenguer *et al*., 2006), as well as transcripts encoding key enzymes of the butyrate formation pathway (acetyl-CoA acetyltransferase, 3-hydroxybutyryl-CoA and butyryl-CoA dehydrogenase, data not shown) (Louis and Flint, 2009; Schwab *et al*., 2014), strongly suggesting the formation of butyrate in the murine forestomach. Lactate cross-feeding might likewise be responsible for the presence of *Desulfovibrionales*, which metabolize lactate and pyruvate to acetate and CO_2_ in the presence of sulfate (Odom and Singleton, 1993); however, too few transcripts of *Desulfovibrionales* prevented a verification of this speculation.

### Extracellular proteins involved in adhesion and biofilm formation

The genomes of *L. reuteri* and *L. johnsonii* harbour many genes encoding large cell surface proteins putatively involved in adhesion to the epithelium and biofilm formation (Pridmore *et al*., 2004; Walter *et al*., 2005; Frese *et al*., 2011). In the forestomach microbiota, transcripts encoding extracellular bacterial surface layer proteins (SLAP, Boot *et al*., 1995) and proteins involved in mucus binding (MucBP and Rib) were significantly more abundant than in the hindgut. MucBP and Rib were most similar to extracellular surface proteins of *L. reuteri* and *L. johnsonii* (for, e.g., ZP_03073480. ZP_12484427, AAT98629, > 90% amino acid sequence identity). *In vitro* and *in vivo* experiments previously confirmed the impact of extracellular proteins in adhesion and biofilm formation. The cell surface protein MucBP of *L. reuteri* ATCC55368 adhered to mucus from different sources (Roos and Jonsson, 2002). Inactivation of a large surface protein (Lsp) of *L. reuteri* 100-23 reduced competitiveness *in vivo* (Walter *et al*., 2005).

Additionally, transcripts of proteins with GH68 and GH70 motives of fructan- and glucansucrases, respectively, were significantly more abundant in forestomach biofilm than in the hindgut lumen microbiota. Proteins with GH68 motive had the highest similarity to *L. reuteri* inulosucrase (CAL25302) and *L. johnsonii* fructosyltransferase (YP_005862512, > 94% amino acid sequence identity). Proteins with GH70 motive indicative of glucansucrases were most similar to glucansucrases of *L. reuteri* (AAU8004 and ABP88725, > 98% amino acid sequence identity). In analogy to the glycansucrase-dependent biofilm formation of *Streptococcus mutans* on the tooth surface, lactobacilli glycansucrases were imposed to be involved in forestomach biofilm formation (Walter *et al*., 2011; Gänzle and Schwab, 2009a). Strengthening this suggestion, reduced competitiveness was reported in two strains of *L. reuteri* after inactivation of extracellular fructansucrases (Walter *et al*., 2011; Sims *et al*., 2011). Similar to *L. reuteri* and *L. johnsonii*, *L. vaginalis* and *L. pontis* possess extracellular fructansucrases (GH68) (Table S4, Tieking *et al*., 2003), which might be one reason for their presence in the forestomach biofilm.

### Protective mechanisms in the forestomach biofilm microbiota

The murine forestomach is an open environment, and bacteria are constantly challenged by acidic pH values, varying oxygen levels and the presence of urea derived from the stomach compartment. Significantly higher abundance of transcripts related to ‘acid stress’ in the forestomach compared with hindgut (1.05 ± 0.82% versus 0.08 ± 0.06%, *P* < 0.05) indicated the prevailing low pH conditions. However, the biofilm community appears to be well adapted to life in this environmental niche, indicated by transcription of genes encoding a diverse array of pathways enhancing stress tolerance via the maintenance of intracellular pH homeostasis, by alterations of the environmental milieu and changes in cell wall composition (Table 1). For instance, major differences between the gene expression profiles in forestomach and hindgut lumen were observed for transcripts assigned to ‘amino acids and derivatives’ (Fig. 4B) and its subcategory ‘arginine; urea cycle, polyamines’. Latter predominantly contained transcripts assigned to ‘urea decomposition’ (38–89% of transcripts of ‘arginine, urea cycle, polyamines) and ‘arginine and ornithine degradation’ (6–38% of transcripts of ‘arginine, urea cycle, polyamines). The degradation of urea enhances acid resistance and facilitates bacterial life in an acidic milieu, such as the stomach. The human stomach colonizer *Helicobacter pylori* depends on its pH regulated urea transporters and an internal urease during colonization of the stomach to maintain a favourable periplasmic pH (Sachs *et al*., 2003). Among strains of *L. reuteri*, urease production is a unique trait of rodent and cereal isolates (Walter *et al*., 2011; Su *et al*., 2012); urea degradation was confirmed using *L. reuteri* sourdough and mouse isolates (Su *et al*., 2012). The importance of urea degradation in forestomach biofilms was highlighted by the transcription of the complete operon for urea uptake and degradation by *L. reuteri* (> 97% amino acid sequence identity, Table 1). In addition, high transcription of the SEED category ‘arginine and ornithine degradation’ encompassing transcripts encoding the arginine deiminase pathway and the arginine/ornithine antiporter ArcD (Fig. 4B, Table 1) indicated another pathway to generate intracellular ammonia from arginine to maintain a neutral intracellular pH in acidic environment (Rollan *et al*., 2003; Su *et al*., 2011).

The decarboxylation of glutamate, which is likely derived from glutamine by the activity of a glutaminase (Su *et al*., 2011), to γ-aminobutyrate (GABA) likewise enhances bacterial acid tolerance. In the forestomach biofilms, genes for glutamate decarboxylase and glutaminase were highly expressed (Table 1). In the rodent isolate *L. reuteri* 100-23, a glutaminase gene is located adjacent to glutamate decarboxylase (Su *et al*., 2011). Inactivation of the glutamate decarboxylase decreased acid resistance as well as competitiveness in sourdoughs (Su *et al*., 2011). As *L. reuteri* 100-23 persists well in both the intestinal tract and cereal fermentations, the impact of this glutamate decarboxylase on acid resistance can be likely expanded to its intestinal habitat.

Biofilm lactobacilli also highly transcribed genes encoding proteins involved in glutathione synthesis, such as glutamate-cysteine ligase, glutathione synthetase and peroxidase (Table 1). The bacterial cell is able to regulate its oxidative state through the conversion of oxidized and reduced forms of glutathione (GSSG and GSH, respectively) catalysed by glutathione reductase and glutathione peroxidase respectively (Pophaly *et al*., 2012). The inactivation of a glutathione reductase impaired oxygen tolerance in *L. sanfranciscensis* (Jänsch *et al*., 2007).

Beside the transcription of pathways increasing acid resistance or altering the oxidative state of the cell, we also observed expression of pathways modifying the structure of the bacterial cell wall. The *dlt* operon is responsible for the integration of d-alanine into cell wall teichoic and lipoteichoic acids, and has been correlated to a number of features, including acid resistance (Boyd *et al*., 2000; Kristian *et al*., 2005). Dlt domains were significantly higher expressed in the forestomach than in the hindgut. Previously, Walter and colleagues (2007) inactivated the *dltA* gene of *L. reuteri* and consequently observed reduced competitiveness *in vivo*; however, adherence was not affected. In contrast to the high transcription of *dtlA* and the glutathione reductase gene observed in the forestomach, *L. plantarum* downregulated *dltA* and the glutathione reductase in the caecum (Marco *et al*., 2009). This observation points again at the forestomach specific adaption of members of the autochthonous biofilm community.

The high expression of a cyclopropane-fatty-acyl-phospholipid synthase indicates modification of the cell membrane. Cyclopropane-fatty-acyl-phospholipid synthase confers unsaturated fatty acids into cyclopropane derivatives leading to enhanced acid tolerance (Brown *et al*., 1997). In *L. brevis*, the expression of a cyclopropane-fatty-acyl-phospholipid synthase is increased during growth in acidic conditions, leading to chances in membrane composition (Behr *et al*., 2006; 2007).

## Conclusions

**Fig. 5:**
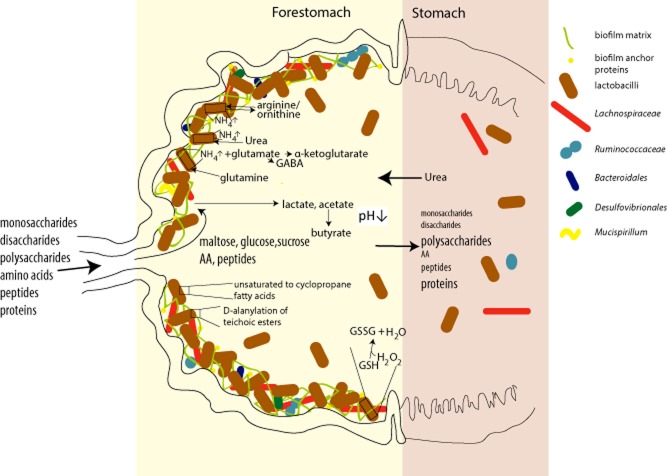
Biofilm lifestyle in the murine forestomach. Schematic diagram depicting the forestomach biofilm community, its substrate utilization and metabolite formation, and mechanism enhancing stress tolerance. Intracellular maintenance of neutral pH (activity of urease, arginine and glutamate metabolism), alterations of the environmental milieu [extracellular protonation of GABA (γ-aminobutyrate or ornithine)] and changes in cell wall composition (d-alanylation of teichoic esters, synthesis of cyclopropane fatty acids) promote acid tolerance. Glutathione contributes to oxygen tolerance of bacterial cells. AA, amino acids; GSH, reduced glutathione; GSSG, oxidized glutathione.

Our study revealed via analysis of *in situ* gene expression the existence of a complex autochthonous forestomach biofilm community interacting via an intrinsic network of functional and metabolic features (Fig. 5). This study confirmed *in situ* the importance of previously identified genes encoding proteins that were shown to be involved in the adaption of strains of *L. reuteri* to the biofilm community (urease, extracellular proteins such as MucBP and GH68 and 70, DltA). Interestingly, transcripts for proteins, such as glutamate decarboxylase and glutathione reductase that assured competitiveness of *L. reuteri* in cereal fermentations, were also highly transcribed in the biofilms, strengthening again the proposed shared intestinal origin of rodent and sourdough isolates (Su *et al*., 2012). In contrast, some genes that were upregulated (copper binding ATPase, IgA protease, sugar PTS systems) during transit of *L. plantarum* and non-rodent *L. johnsonii* through the murine GIT (Bron *et al*., 2004; Denou *et al*., 2007) were not identified as significantly increased in the forestomach, while others were differentially regulated (*dltA*, glutathione reductase) (Marco *et al*., 2009) in line with variations in lifestyle of the *Lactobacillus* species (transit versus persistence) and varying environmental parameters in forestomach and hindgut (substrate availability, pH and oxygen levels). These observations strengthen the importance of considering habitat adaption when applying strains of *Lactobacillus* for health application, e.g. as probiotics. In addition, as comparable lactobacilli-dominated biofilm communities exist in pigs, horses, birds and the human vagina, results obtained here might be exemplary for other autochthonous *Lactobacillaceae* biofilms.

## Experimental procedures

### Animals

C57BL/6 mice of 6–8 weeks of age were sacrificed, the stomach was separated from the other parts of the gastrointestinal system, stomach contents were removed, and the forestomach was snap-frozen. For nucleic acid (NA) isolation of the forestomach biofilm, bacterial cells were recovered from the forestomach epithelium by scratching and flushing with 0.5 ml anoxic PBS, and bacterial cells were collected by centrifugation and immediately used for NA isolation. For NA isolation from the hindgut, caecum and colon were flushed together with 7 ml of PBS, flushed hindgut contents were homogenized, collected by centrifugation and snap-frozen for NA purification (Berry *et al*., 2012). Animal experiments were approved by the institutional ethics committee and conducted in accordance with protocols approved by the Austrian laws (BMWF-66.006/0002-II/10b/2010). FS1-3 and hindguts (CL1-3, IL1-3) were obtained from animals housed at a different facility than animals of FS4 and FS5. Samples CL1-3 and IL1-3 were derived from previous studies (Schwab *et al*., 2014).

### NA isolation and purification of RNA and DNA and cDNA synthesis

DNA and RNA were extracted with a phenol-chloroform bead-beating procedure and kit purification (Qiagen AllPrep DNA/RNA Mini kit) as previously described (Berry *et al*., 2012). Total RNA was reverse-transcribed using the SuperScript Double-Stranded cDNA Synthesis Kit (Invitrogen) with modifications as described before (Berry *et al*., 2012).

### Sequencing of cDNA libraries

cDNA libraries generated from total RNA derived from the forestomach were sequenced by a PGM IonTorrent (LifeSciences) using 200 bp sequencing chemistry and 314, 316 or 318 chips according to instructions supplied by the manufacturer. cDNA libraries were size-selected (> 100 bp) before further processing. Double-stranded cDNA libraries from the hindgut were paired-end sequenced using an Illumina HiSeq (CSF Vienna) and were overlapped using FLASH (Magoč and Salzberg, 2011) as described before (Schwab *et al*., 2014).

### Metatranscriptome data analysis

Metatranscriptomic sequencing data were analysed following the double RNA analysis pipeline utilized by Urich and colleagues (2008). Community composition was determined from 100 000 rRNA reads, which were taxonomically assigned using CREST (Urich *et al*., 2008; Lanzén *et al*., 2012) (bit score = 150, top percent = 10, minimal support = 1). mRNA tags were compared against the NCBI RefSeq database using BlastX, and functionally and taxonomically classified using MEGAN and the SEED functional classification scheme therein (bit score = 40, top percent = 10, minimal support = 1) (Mitra *et al*., 2011). This resulted in between 7331 and 26 794 and up to 214 654 functionally annotated mRNAs for IonTorrent or Illumina derived samples respectively (Table S1). Metatranscriptomes were generated from five forestomachs and six hindgut contents from caecum and colon. Metatranscriptome data IL1-3 were also used in a previous manuscript with a different topic (Schwab *et al*., 2014). past (Hammer *et al*., 2001) was used for multivariate analysis of metatranscriptome data. Principal component analysis was done by eigenvalue decomposition of a data variance-covariance matrix. Unpaired t-test analysis in SigmaPlot 11 (Systat) was applied for statistical analysis of variance. Metatranscriptomic data is deposited at NCBI's Sequence Read Archive under accession numbers SRP026649, SRP026292 and SRP027343.

### *Lactobacillaceae* community composition in biofilm and forestomach

In order to investigate the composition of the *Lactobacillaceae* community of forestomach and the caecum/colon, 16S rRNA transcripts extracted from MEGAN were compared against a custom-made reference database containing 153 *Lactobacillus*-type strains obtained from the SILVA database (Quast *et al*., 2013) using CREST (BlastN, minimum bit score = 150, top percent = 1, minimal support = 20 and 50 for hindgut and forestomach derived samples respectively). To verify that assignment at the species level was possible in spite of the short 16S rRNA read length, we performed a simulation using randomly generated 16S rRNA fragments of *Lactobacillus* strains identified in our study. We split the 16S rRNA of *L. vaginalis*, *L. fermentum*, *L. reuteri*, *L. oris*, *L. pontis*, *L. frumenti*, *L. antri*, *L. intestinal*, *L. johnsonii* and *L. taiwanensis* in *n* = 500 fragments of 170 bp, and compared those fragments against our *Lactobacillus-*type strain database using BlastN. All strains were accurately assigned to the corresponding type strains with highest recovery rates for *L. reuteri*, *L. fermentum* and *L. vaginalis* (> 80%), medium recovery rates for *L. pontis*, *L.intestinalis* and *L. johnsonii* (68–50%), and low recovery rates for *L. oris*, *L. frumenti*, *L. antri* (47–33%) and *L. taiwanensis* (17%). No false-positive assignment (i.e. assignment of fragment to wrong species) was observed. This simulation, verified the validity of our approach. Due to the low recovery rates reads of *L. oris*, *L. frumenti* and *L. antri* were consequently only assigned at the genus level.

As the *L. johnsonii* and *L. taiwanensis* 16S rRNA genes are highly similar (99.5%, Wang *et al*., 2009), we omitted *L. taiwanensis* from our type strain database. This resulted in the improved recovery of 83% of the reads of *L. taiwanensis* to *L. johnsonii/taiwanensis* reference sequence in the simulation. We then compared all 16S rRNA *Lactobacillaceae* transcripts extracted from MEGAN against the modified type strain reference database (Fig. 2).

### Pfam analysis

mRNA reads were translated into all six frames, each frame into separate open reading frames (ORFs), avoiding any ‘*’ characters marking stop codons in a resulting ORF. All ORFs equal to 30 amino acids or larger were screened for assignable conserved protein domains using reference HMMs (hidden Markov models) of the Pfam database (Punta *et al*., 2012; Pfam release 25, http://Pfam.janelia.org) with hmmer tools (http://hmmer.janelia.org/). All database hits with *e*-values below a threshold of 10^−4^ were counted. Translated reads of interest were subjected to blastp searches against NCBI's nr database. Due to the different sizes of metatranscriptomes derived from IonTorrent and Illumina sequencing (Table S1), we focused on Pfam categories that were significantly higher transcribed in IonTorrent-derived metatranscriptomes.

### Quantification of selected 16S rRNA genes by quantitative PCR (qPCR)

Copies of 16S rRNA genes were quantified by qPCR using a Mastercycler ep realplex (Eppendorf). Reaction mixtures (20 μl) contained 10 μl QuantiFast SybrGreen (Qiagen), 1 μl of each of the specific primers (Table S2) at a final concentration of 0.25 μ M, and 1 μl of template DNA. Running conditions were 95°C for 3 min followed by 40 cycles of 95°C for 10 s, annealing for 15 s at 60°C, and 72°C for 30 s. Melting curve analysis and agarose gel electrophoresis were performed to verify the identity of the genes of interest. Samples were run in duplicates. Standard curves were generated from linearized plasmids. Gene copies were calculated per μg DNA.

## Supporting information

**Fig. S1.** Relative abundance of all transcripts assigned to major SEED categories that were recovered from forestomachs and hindguts (A), and (B) relative abundance of transcripts of *Lactobacillales* and *Clostridiales* in forestomachs and hindguts. From the hindguts, we obtained enough mRNA transcripts for functional analysis (*n* > 400) from only two samples.

**Fig. S2.** Host niche-dependent gene expression. Principal component analysis based on relative abundance of SEED categories in the forestomach and the hindgut of either the entire community (hindgut, forestomach), or of *Lactobacillales* and *Clostridiales* residing in forestomach or hindgut. As only two hindgut samples of the six C57BL/6 investigated yielded enough *Lactobacillales* mRNA reads for functional analysis, we strengthened our analysis including two additional samples obtained from Tyk2^-/-^ mice on a C57BL/6 background with *n* = 1504 and *n* = 1071 *Lactobacillales* mRNA reads.

**Table S1.** Metatranscriptome sequencing. Forestomach metatranscriptomes were sequenced using an IonTorrent PGM sequencer. Double-stranded cDNA libraries derived from the hindgut were paired-end sequenced using an Illumina HiSeq (Campus Science Support Facilities GmbH, Vienna). Read pairs were overlapped using FLASH (Magoč and Salzberg, 2011). Metatranscriptomic sequencing data were analysed following an established double RNA analysis pipeline (Urich *et al*., 2008; Berry *et al*., 2012).

**Table S2.** Quantitative PCR of 16S rRNA gene of lactic acid bacteria and *Clostridium* clusters IV and XIV. Shown are 16S rRNA gene copies μg^−1^ DNA of individual forestomachs (FS1-5) and the mean of six hindgut samples. To determine the gene copies of 16S rRNA genes of *Clostridium* clusters IV and XIVa, primers targeting the respective clusters were used in separate reactions, gene copies were added and the sum was logarithmized.

**Table S3.** Major glycoside hydrolase (GH) families in forestomach and hindgut according to Pfam analysis.

**Table S4.** Selected features of the genome of *L. vaginalis* ATCC 49540 (NZ_GG693412).

## References

[b1] Behr J, Gänzle MG, Vogel RF (2006). Characterization of a highly hop-resistant *Lactobacillus brevis* strain lacking hop transport. Appl Environ Microbiol.

[b2] Behr J, Gänzle MG, Vogel RF (2007). Proteomic approach for characterization of hop-inducible proteins in *Lactobacillus brevis*. Appl Environ Microbiol.

[b3] Belenguer A, Duncan SH, Calder AG, Holtrop G, Louis P, Lobley GE, Flint HJ (2006). Two routes of metabolic cross-feeding between *Bifidobacterium adolescentis* and butyrate-producing anaerobes from the human gut. Appl Environ Microbiol.

[b4] Berry D, Schwab C, Milinovich G, Reichert J, Ben Mahfoudh K, Decker T (2012). Phylotype-level 16S rRNA analysis reveals new bacterial indicators of health state in acute murine colitis. ISME J.

[b5] Boot HJ, Kolen CP, Pouwels PH (1995). Identification, cloning, and nucleotide sequence of a silent S-layer protein gene of *Lactobacillus acidophilus* ATCC 4356 which has extensive similarity with the S-layer protein gene of this species. J Bacteriol.

[b6] Boyd DA, Cvitkovitch DG, Bleiweis AS, Kiriukhin MY, Debabov DV, Neuhaus FC (2000). Defects in D-alanyl-lipoteichoic acid synthesis in *Streptococcus mutans* results in acid sensitivity. J Bacteriol.

[b7] Bron PA, Grangette C, Mercenier A, de Vos WM, Kleerebezem M (2004). Identification of *Lactobacillus plantarum* genes that are induced in the gastrointestinal tract of mice. J Bacteriol.

[b8] Brown JL, Ross T, McMeekin TA, Nichols PD (1997). Acid habituation of *Escherichia coli* and the potential role of cyclopropane fatty acids in low pH tolerance. Int J Food Microbiol.

[b9] Buhnik-Rosenblau K, Matsko-Efimov V, Jung M, Shin H, Danin-Poleg Y, Kashi Y (2012). Indication for co-evolution of *Lactobacillus johnsonii* with its hosts. BMC Microbiol.

[b10] Canchaya C, Claesson MJ, Fitzgerald GF, van Sinderen D, O'Toole PW (2006). Diversity of the genus *Lactobacillus* revealed by comparative genomics of five species. Microbiology.

[b11] Claesson MJ, van Sinderen D, O'Toole PW (2007). The genus *Lactobacillus*: a genomic basis for understanding its diversity. FEMS Microbiol Lett.

[b12] Denou E, Berger B, Barretto C, Panoff J-M, Arigoni F, Brüssow H (2007). Gene expression of commensal *Lactobacillus johnsonii* strain NCC533 during in vitro growth and in the murine gut. J Bacteriol.

[b13] Denou E, Pridmore RD, Berger B, Panoff J-M, Arigoni F, Brüssow H (2008). Identification of genes associated with long-gut persistence phenotype of the probiotic *Lactobacillus johnsonii* strain NCC533 using a combination of genomics and transcriptome analysis. J Bacteriol.

[b14] Duncan SH, Louis P, Flint HJ (2004). Lactate-utilizing bacteria, isolated from human feces, that produce butyrate as major fermentation product. Appl Environ Microbiol.

[b15] Embley TM, Faquir N, Bossart W, Collins MD (1989). *Lactobacillus vaginalis* sp. nov. from the human vagina. Int J Syst Bacteriol.

[b16] Felis GE, Dellaglio F (2007). Taxonomy of lactobacilli and bifidobacteria. Curr Issues Intest Microbiol.

[b17] Frese SA, Benson SK, Tannock GW, Loach DM, Kim J, Zhang M (2011). The evolution of host specialization of the vertebrate gut symbiont *Lactobacillus reuteri*. PLoS Genet.

[b18] Fuller R, Barrow PA, Brooker BE (1978). Bacteria associated with the gastric epithelium of neonatal pigs. Appl Environ Microbiol.

[b19] Gänzle M, Schwab C, Arendt E, Dal Bello F (2009b). Exploitation of the metabolic potential of lactic acid bacteria for improved quality of gluten-free bread. The Science of Gluten-Free Foods and Beverages.

[b20] Gänzle MG, Follador R (2012). Metabolism of oligosaccharides and starch in lactobacilli: a review. Front Microbiol.

[b21] Gänzle MG, Schwab C, Ullrich M (2009a). Ecology of exopolysaccharide formation by lactic acid bacteria: sucrose utilisation, stress tolerance, and biofilm formation. Bacterial Polysaccharides: Current Innovations and Future Trends.

[b22] Gänzle MG, Vermeulen N, Vogel RF (2007). Carbohydrate, peptide and lipid metabolism of lactic acid bacteria in sourdough. Food Microbiol.

[b23] Guinane CM, Kent RM, Norberg S, Hill C, Fitzgerald GF, Stanton C (2011). Host specific diversity in *Lactobacillus johnsonii* as evidenced by a major chromosomal inversion and phage resistance mechanism. PLoS ONE.

[b24] Hammer Ø, Harper DAT, Ryan PD (2001). PAST: paleontological statistics software package for education and data analysis. Palaeontol Electron.

[b25] Helbling DE, Ackermann M, Fenner K, Kohler H-PE, Johnson DR (2012). The activity level of a microbial community function can be predicted from its metatranscriptome. ISME J.

[b26] Jänsch A, Korakli M, Vogel RF, Gänzle MG (2007). Glutathione reductase from *Lactobacillus sanfranciscensis* DSM20451T: contribution to oxygen tolerance and thiol exchange reactions in wheat sourdough. Appl Environ Microbiol.

[b27] Kristian SA, Datta V, Weidenmaier C, Kansal R, Fedtke I, Peschel A (2005). D-alanylation of teichoic acids promotes group A *Streptococcus* antimicrobial peptide resistance, neutrophil survival, and epithelial cell invasion. J Bacteriol.

[b28] Lanzén A, Jørgensen SL, Huson DH, Gorfer M, Grindhaug SH, Jonassen I (2012). CREST – Classification Resources for Environmental Sequence Tags. PLoS ONE.

[b29] Louis P, Flint HJ (2009). Diversity, metabolism and microbial ecology of butyrate-producing bacteria from the human large intestine. FEMS Microbiol Lett.

[b30] Magoč T, Salzberg SL (2011). FLASH: fast length adjustment of short reads to improve genome assemblies. Bioinformatics.

[b31] Marco ML, Bongers RS, de Vos WM, Kleerebezem M (2007). Spatial and temporal expression of *Lactobacillus plantarum* genes in the gastrointestinal tract of mice. Appl Environ Microbiol.

[b32] Marco ML, Peters THF, Bongers RS, Molenaar D, van Hemert S, Sonnenburg JL (2009). Lifestyle of *Lactobacillus plantarum* in the mouse caecum. Environ Microbiol.

[b33] Mitra S, Stärk M, Huson DH (2011). Analysis of 16S rRNA environmental sequences using MEGAN. BMC Genomics.

[b34] Odom JM, Singleton R (1993). The Sulfate-Reducing Bacteria: Contemporary Perspectives.

[b35] Oh PL, Benson AK, Peterson DA, Patil PB, Moriyama EN, Roos S (2010). Diversification of the gut symbiont *Lactobacillus reuteri* as a result of host-driven evolution. ISME J.

[b36] Pophaly SD, Singh R, Pophaly SD, Kaushik JK, Tomar SK (2012). Current status and emerging role of glutathione in food grade lactic acid bacteria. Microb Cell Fact.

[b37] Pridmore RD, Berger B, Desiere F, Vilanova D, Barretto C, Pittet A-C (2004). The genome sequence of the probiotic intestinal bacterium *Lactobacillus johnsonii* NCC 533. PNAS.

[b38] Punta M, Coggill PC, Eberhardt RY, Mistry J, Tate J, Boursnell C (2012). The Pfam protein families database. Nucleic Acids Res.

[b39] Quast C, Pruesse E, Yilmaz P, Gerken J, Schweer T, Yarza P (2013). The SILVA ribosomal RNA gene database project: improved data processing and web-based tools. Nucleic Acids Res.

[b40] Rollan G, Lorca GL, Font de Valdez G (2003). Arginine catabolism and acid tolerance response in *Lactobacillus reuteri* isolated from sourdough. Food Microbiol.

[b41] Roos S, Jonsson H (2002). A high-molecular-mass cell-surface protein from *Lactobacillus reuteri* 1063 adheres to mucus components. Microbiology.

[b42] Sachs G, Weeks DL, Melchers K, Scott DR (2003). The gastric biology of *Heliobacter pylori*. Annu Rev Physiol.

[b43] Savage DC (1972). Associations and physiological interactions of indigenous microorganisms and gastrointestinal epithelia. Am J Clin Nutr.

[b44] Schwab C, Berry D, Rauch I, Rennisch I, Ramesmayer J, Hainzl E (2014). Longitudinal study of murine microbiota activity and interactions with the host during acute inflammation and recovery. ISME J.

[b45] Sims IM, Frese SA, Walter J, Loach D, Wilson M, Appleyard K (2011). Structure and functions of exopolysaccharide produced by gut commensal *Lactobacillus reuteri* 100-23. ISME J.

[b46] Su MS-W, Schlicht S, Gänzle MG (2011). Contribution of glutamate decarboxylase in *Lactobacillus reuteri* to acid resistance and persistence in sourdough. Microb Cell Fact.

[b47] Su MS-W, Phaik LO, Walter J, Gänzle MG (2012). Phylogenetic, genetic, and physiological analysis of sourdough isolates of *Lactobacillus reuteri:* food fermenting strains of intestinal origin. Appl Environ Microbiol.

[b48] Tannock GW, Crichton C, Welling GW, Koopman JP, Midtvedt T (1988). Reconstitution of the gastrointestinal microflora of lactobacillus-free mice. Appl Environ Microbiol.

[b49] Tannock GW, Wilson CM, Loach D, Cook GM, Eason J, O'Toole PW (2012). Resource partitioning in relation to cohabitation of *Lactobacillus* species in the mouse forestomach. ISME J.

[b50] Tieking M, Korakli M, Ehrmann MA, Gänzle MG, Vogel RF (2003). *In situ* production of exopolysacchardes during sourdough fermentation by cereal and intestinal isolates of lactic acid bacteria. Appl Environ Microbiol.

[b51] Urich T, Lanzén A, Qi J, Huson DH, Schleper C, Schuster SC (2008). Simultaneous assessment of soil microbial community structure and function through analysis of the meta-transcriptome. PLoS ONE.

[b52] Vogel RF, Böcker G, Stolz P, Ehrmann M, Fanta D, Ludwig W (1994). Identification of lactobacilli from sourdough and description of *Lactobacillus pontis* sp. nov. Int J Syst Bacteriol.

[b53] Walter J (2008). Ecological role of lactobacilli in the gastrointestinal tract: implications for fundamental and biomedical research. Appl Environ Microbiol.

[b54] Walter J, Britton RA, Roos S (2011). Host-microbial symbiosis in the vertebrate gastrointestinal tract and the *Lactobacillus reuteri* paradigm. PNAS.

[b55] Walter J, Schwab C, Loach DM, Ganzle MG, Tannock GW (2008). Glucosyltransferase A (GtfA) and inulosucrase (Inu) of *Lactobacillus reuteri* TMW1.106 contribute to cell aggregation, in vitro biofilm formation, and colonization of the mouse gastrointestinal tract. Microbiology.

[b56] Walter J, Chagnaud P, Tannock GW, Loach DM, Dal Bello F, Jenkinson HF (2005). A high-molecular-mass surface protein (Lsp) and methionine sulfoxide reductase B (MsrB) contribute to the ecological performance of *Lactobacillus reuteri* in the murine gut. Appl Environ Microbiol.

[b57] Walter J, Loach DM, Alqumber M, Rockel C, Hermann C, Pfitzenmaier M (2007). D-alanyl ester depletion of teichoic acids in *Lactobacillus reuteri* 100-23 results in impaired colonization of the mouse gastrointestinal tract. Environ Microbiol.

[b58] Wang LT, Kuo HP, Wu YC, Tai CJ, Lee FL (2009). *Lactobacillus taiwanensis sp. nov*, isolated from silage. Int J Syst Evol Microbiol.

[b59] Yuki N, Shimazaki T, Kushiro A, Watanabe K, Uchida K, Yuyama T (2000). Colonization of the stratified squamous epithelium of the nonsecreting area of horse stomach by lactobacilli. Appl Environ Microbiol.

