# Supplementary material for: Gene expression of lactobacilli in murine forestomach biofilms
Source: Microb Biotechnol. 2014 Apr 4;7(4):347–59. doi: 10.1111/1751-7915.12126 (PMC4241727; doi:10.1111/1751-7915.12126)
Supplement: Supplementary file 1 [file mbt20007-0347-sd1.doc]

Supplementary information

**Gene expression of lactobacilli in murine forestomach biofilms**

Clarissa Schwab^1^*, Alexander Tøsdal Tveit^2^, Christa Schleper^1^, Tim Urich^1^

^1^University of Vienna, Division of Archaea Biology and Ecogenomics, Department of Ecogenomics and Systems Biology, Althanstrasse 14, 1090 Wien, Austria

^2^ University of Tromsø, Department of Arctic and Marine Biology, 9037 Tromsø, Norway

^*^Corresponding author: Clarissa Schwab, current address: Laboratory of Food Biotechnology, Institute of Food, Nutrition and Health, ETH Zürich, Schmelzbergstrasse 7, 8092 Switzerland, Tel: +41 4225371, Email: clarissa.schwab@hest.ethz.ch)

**Titles and legends to supplementary figures**

Figure S1. Relative abundance of all transcripts assigned to major SEED categories that were recovered from forestomachs and hindguts (A), and (B) relative abundance of transcripts of *Lactobacillales* and *Clostridiales* in forestomachs and hindguts. From the hindguts, we obtained enough mRNA transcripts for functional analysis (n>400) from only 2 samples.

**Figure S2.** Host niche dependent gene expression. Principal component analysis based on relative abundance of SEED categories in the forestomach and the hindgut of either the entire community (hindgut, forestomach) or of *Lactobacillales* and *Clostridiales* residing in forestomach or hindgut. As only two hindgut samples of the six C57BL/6 investigated yielded enough *Lactobacillales* mRNA reads for functional analysis, we strengthened our analysis including two additional samples obtained from Tyk2^-/-^ mice on a C57BL/6 background with n=1504 and n=1071 *Lactobacillales* mRNA reads (Figure S2).

**Figure S1.**

**Figure S2.**

**Table S1**. Metatranscriptome sequencing. Forestomach metatranscriptomes were sequencing using an IonTorrent PGM sequencer. Double stranded cDNA libraries derived from the hindgut were paired-end sequenced using an Illumina HiSeq (Campus Science Support Facilities GmbH, Vienna). Read pairs were overlapped using FLASH (Magoč and Salzberg, 2011). Metatranscriptomic sequencing data were analyzed following an established double RNA analysis pipeline (Berry *et al.*, 2012; Urich *et al.*, 2008).

| **Location** | **Sample ID** | **Fasta reads** | **Average length (bp)** | **rRNA reads (%)** | **mRNA reads (%)** | **mRNA reads assigned to SEED in Megan (%)** | ***Lactobacillales* mRNA reads (%)** |
| --- | --- | --- | --- | --- | --- | --- | --- |
| Forestomach | FS1 | 1.207.733 | 216 | 1.114.376 (92.3) | 92.857 (7.7) | 26.586 (28.6) | 16.821 (57.3) |
|  | FS2 | 3.045.796 | 233 | 2.997.719 (98.4) | 48.077 (1.6) | 23.490 (48.9) | 20.467 (87.1) |
|  | FS3 | 526.212 | 212 | 503.957(95.8) | 22.255 (4.2) | 7331 (32.9) | 4664 (63.3) |
|  | FS4 | 2.590.980 | 203 | 2.542.692 (98.1) | 48.288 (1.7) | 18.269 (37.8) | 2507 (13.7) |
|  | FS5 | 4.756.611 | 181 | 4.612.157 (97.0) | 144.454 (3.0) | 26.794 (18.6) | 11.861 (44.3) |
| Hindgut | IL1 | 5.126.605 | 166 | 4.892.326 (95.4) | 234.279 (4.6) | 82.315 (35.1) | <150 |
|  | IL2 | 8.377.912 | 167 | 7.839.163 (93.6) | 538.749 (6.4) | 177.048 (32.9) | <150 |
|  | IL3 | 4.173.904 | 156 | 3.979.306 (95.3) | 194.598 (4.7) | 57.939 (29.8) | <150 |
|  | CL1 | 13.298.688 | 163 | 12.542.952 (94.3) | 755.736 (5.7) | 214.654 (28.4) | 1194 (0.6) |
|  | CL2 | 10.508.499 | 160 | 10.763.925 (97.6) | 255.426 (2.4) | 72.963 (28.6) | 407 (0.6) |
|  | CL3 | 3.238.827 | 161 | 3.108.145 (95.8) | 130.682 (4.2) | 32.071 (24.5) | <150 |

**Table S2.** Quantitative PCR of 16S rRNA gene of lactic acid bacteria and *Clostridium* clusters IV and XIV. Shown are 16S rRNA gene copies µg^-1^ DNA of individual forestomachs (FS1-5) and the mean of six hindgut samples (Lumen). To determine the gene copies of 16S rRNA genes of *Clostridium* clusters IV and XIVa, primers targeting the respective clusters were used in separate reactions, gene copies were added and the sum was logarithmised.

| **Sample ID** | **Lactic acid bacteria^1^** | ***Clostridium* clusters IV and XIVa^2,3^** |
| --- | --- | --- |
|  | 16S rRNA gene copies µg^-1^ DNA | |
| FS1 | 8.47 | 7.07 |
| FS2 | 8.14 | 7.08 |
| FS3 | 8.75 | 7.28 |
| FS4 | 7.92 | 7.92 |
| FS5 | 8.20 | 6.65 |
| Lumen | 6.76±0.27 | 8.35±0.43 |

Primers used:^1^Lactic acid bacteria forward 5’-AGCAGTAGGGAATCTTCCA-3’, reverse 5’-CACCGCTACACATGGAG-3’, ^2^*Clostridium leptum* – *Fecalibacterium prausnitzii* subgroup (*Clostridium* genus cluster IV) (forward 5’-GCACAAGCAGTGGAGT-3’, reverse 5’-CTTCCTCCGTTTTGTCAA-3’), ^3^*Clostridium coccoides* – *Eubacterium rectale* subgroup (*Clostridium* cluster XIVa and XIVb) (forward 5’-AAATGACGGTACCTGACTAA-3’, reverse 5’- TTTGAGTTTCATTCTTGCGAA-3’) as summarized in Schwab *et al.*, 2012.

**Table S3. Major glycoside hydrolase (GH) families in forestomach and hindgut according to Pfam analysis.**

| **Forestomach** |  |  | **Hindgut** |  |  |
| --- | --- | --- | --- | --- | --- |
| **GH family** | **Predicted function** | **% relative abundance** | **GH family** | **Predicted function** | **% relative abundance** |
| Glyco_hydro_65M | Maltose phosphorylase | 0.168±0.111 | Glyco_hydro_48 | Endocellobiohydrolase | 0.161±0.141 |
| Glyco_hydro_1 | Beta-glucosidase | 0.100±0.054 | Glyco_hydro_3 | Beta-glucosidase | 0.131±0.030 |
| Glyco_hydro_65N | Maltose phosphorylase | 0.068±0.064 | Glyco_hydro_77 | Amylomaltase | 0.090±0.014 |
| Glyco_hydro_31 | Alpha-glucosidase | 0.055±0.011 | Glyco_hydro_2_C | Beta-galactosidase | 0.089±0.016 |
| Glyco_hydro_65C | Maltose phosphorylase | 0.036±0.043 | Glyco_hydro_9 | Endoglucanase | 0.079±0.052 |
| Glyco_hydro_77 | Amylomaltase | 0.036±0.033 | Glyco_hydro_31 | Alpha-glucosidase | 0.076±0.012 |
| Glyco_hydro_70 | Glucansucrase | 0.034±0.033 | Glyco_hydro_1 | Beta-glucosidase | 0.073±0.036 |
| Glyco_hydro_68 | Fructansucrase | 0.011±0.012 | Glyco_hydro_43 | Beta-xylosidase | 0.064±0.037 |

**Table S4.** Selected features of the genome of *L. vaginalis* ATCC 49540 (NZ_GG693412).

| **Function** | **Gene** | Locus tag (HMPREF0549_) |
| --- | --- | --- |
| Carbohydrate utilization | Maltose phosphorylase | _0996 |
| Amino acid utilization and uptake | Signal peptidase | _1066 |
|  | aminopeptidase | _1092 |
|  | dipeptidase A | _1106 |
|  | Peptidase M13 | _1144 |
|  | Carboxypeptidase | _0984 |
|  | Peptidase S24 | _1164 |
|  | Peptidase C69 | _1186, _1239 |
|  | D-alanyl-D-alanine carboxypeptidase | _1215, _1325 |
|  | prolyl aminopeptidase | _1258 |
|  | x-prolyl-dipeptyl aminopeptidase | _1259 |
|  | Peptidase M10 | _1300 |
|  | peptidase A24A domain protein | _1376 |
|  | methionine aminopeptidase | _1478 |
|  | branched-chain amino acid (ABC) transporter | _0936, _0937, _1249 |
|  | Amino acid permease | _1114, _1220, _1268 |
| Glutamine/Glutamate | glutamine ABC transporter permease | _1352 |
|  | glutamine ABC transporter substrate-binding | _0960 |
|  | glutaminase | _1294 |
|  | glutamate--cysteine ligase | _1467, _0994 |
| Extracellular proteins / biofilm formation | Levansucrase (GH68) | _0954 |
|  | LPXTG-motif cell wall anchor domain protein | _0966 |
|  | mucus binding protein | _1016 |
|  | possible surface protein C | _1519 |

**References**

Berry, D., Schwab, C., Milinovich, G., Reichert, J., Ben Mahfoudh, K., Decker, T., *et al*. (2012) Phylotype-level 16S rRNA analysis reveals new bacterial indicators of health state in acute murine colitis. ISME J 6: 2091-2106.

Magoč, T., and Salzberg, S.L. (2011) FLASH: fast length adjustment of short reads to improve genome assemblies. Bioinformatics 27: 2957-2963.

Schwab, C., and Gänzle, M.G. (2011) Diet and environment shape fecal bacterial microbiota composition and enteric pathogen load of grizzly bears. PLoS ONE 6: e27905.

Urich, T., Lanzén, A., Qi, J., Huson, D.H., Schleper, C., and Schuster, S.C. (2008) Simultaneous assessment of soil microbial community structure and function through analysis of the meta-transcriptome. PLoS ONE 3: e2527.
